# Haematological, Biochemical and Acid‐Base Changes in Uraemic Dogs Undergoing Intermittent Haemodialysis (2017–2024)

**DOI:** 10.1002/vms3.70873

**Published:** 2026-03-05

**Authors:** Diego Ribeiro, Reiner S. de Moraes, Silvano S. Geraldes, Henry D. Mogollón‐García, Paulo F. Marcusso, Alessandra Melchert, Adriano S. Okamoto, Priscylla T. C. Guimarães‐Okamoto

**Affiliations:** ^1^ Department of Veterinary Clinic Faculty of Veterinary Medicine and Zootechny São Paulo State University (UNESP) Botucatu São Paulo Brazil; ^2^ Department of Veterinary Surgery and Animal Reproduction Faculty of Veterinary Medicine and Zootechny São Paulo State University (UNESP) Botucatu São Paulo Brazil

**Keywords:** acid‐base, dialysis, haematology, prevalence, uraemia

## Abstract

Understanding laboratory variables in animals undergoing haemodialysis is essential for optimizing therapeutic strategies. This study investigated the laboratory variables of uraemic crisis dogs undergoing intermittent haemodialysis (IHD). Medical records of dogs with chronic kidney disease (CKD) in uraemic crisis and those with acute kidney injury (AKI) undergoing IHD between 2017 and 2024 were reviewed. Fifty‐eight dogs and 149 sessions were included. A high prevalence of anaemia and hypoalbuminaemia was observed at admission to the IHD, with the prevalence increasing as the number of sessions increased. Among the dogs with AKI and CKD, 84.6% and 78.1%, respectively, had anaemia. Acidaemia, metabolic acidosis and secondary respiratory alkalosis were common and were corrected after the sessions. Among dogs whose pH was within the reference range at admission to IHD, 43.5% exhibited reductions in bicarbonate and PCO_2_. The prevalence of metabolic acidosis was 40.6% in CKD and 34.6% in AKI. After the sessions, there were decreases (*p* ≤ 0.001) in RBC, haematocrit, MCHC, platelets, creatinine, urea, phosphorus, plasma proteins and potassium, and increases (*p* ≤ 0.043) in MCV, pH, HCO_3_, the anion gap, chloride, sodium and ionized calcium. There was an average decrease in haematocrit of 3.42‐5% per session (*p* < 0.0001). The documented laboratory changes support early therapeutic planning. Acid‐base correction and toxin removal highlight the efficacy of IHD, while the progression of anaemia and hypoalbuminaemia requires continued monitoring. Estimating the average decrease in haematocrit per session allows for the prediction of haematocrit decline, enabling more precise planning of priming fluid selection and blood product replacement.

## Graphical abstract

This study investigated the laboratory variables of uraemic crisis dogs undergoing intermittent haemodialysis (IHD). The documented laboratory changes support early therapeutic planning. Acid‐base correction and toxin removal highlight the efficacy of IHD, while the progression of anaemia and hypoalbuminaemia requires continued monitoring. Estimating the average decrease in haematocrit per session allows for the prediction of haematocrit decline, enabling more precise planning of priming fluid selection and blood product replacement.

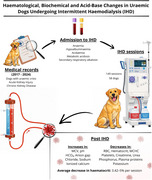

## 1 Introduction

Haemodialysis is an extracorporeal renal replacement therapy used to support the treatment of uraemic crisis in dogs and cats due to acute kidney injury (AKI) or chronic kidney disease (CKD) (Poeppel and Langston 2023). Based on the principles of diffusion, ultrafiltration and convection, this technique helps to correct hydroelectrolytic and acid‐base imbalances and to remove toxins and metabolites, contributing to the restoration of homeostasis (Cowgill 2011; Segev et al. 2024).

Although it is considered a safe therapy, haemodialysis can cause adverse haematological changes, particularly due to blood contact with the extracorporeal circuit and the use of anticoagulants (Panagoutsos et al. 2002; Geraldes et al. 2019; Picelli et al. 2022). These effects are even more relevant in patients with AKI and CKD in uraemic crisis, as the accumulation of toxins, protein losses, acid‐base dysfunction and the inflammatory process resulting from renal parenchymal deterioration promote the development of haematological, biochemical and gasometric disorders (Sonu et al. 2019; Lippi et al. 2021).

Previous studies, such as that by Geraldes et al. (2019) (*n* = 14), reported reductions in red blood cell counts (RBC), platelets and total protein after intermittent haemodialysis (IHD) sessions. In addition, Langston et al. (2017) reported that at least 32% of dogs undergoing renal replacement therapy required blood transfusions throughout the treatment, suggesting possible clinical implications. Therefore, evaluation of haematological changes, serum biochemistry and acid‐base balance upon admission to haemodialysis, along with understanding their contribution to correcting or exacerbating these changes, is essential for more precise clinical management.

This retrospective study aimed to analyze and describe the haematologic variables, serum biochemistry and acid‐base balance of dogs in uraemic crisis on admission to IHD and to evaluate the changes induced by the dialysis sessions in these parameters. The hypotheses were that: (1) there would be a high prevalence of anaemia, hypoalbuminaemia, acidaemia and metabolic acidosis at admission for IHD; (2) reductions would occur in variables such as haematocrit, platelet count, creatinine, urea, phosphorus, plasma proteins and potassium following IHD sessions; and (3) acid–base disorders would be corrected after haemodialysis.

## 2 Materials and Methods

### 2.1 Animal Selection

The medical records of dogs undergoing IHD at the Dialysis Centre of the Veterinary Hospital of the Faculty of Veterinary Medicine and Zootechny (HV‐FMVZ) of São Paulo State University (UNESP—Botucatu) were retrospectively reviewed. The selection criteria included dogs with AKI and CKD in uraemic crisis that underwent IHD between 2017 and 2024. This selection was based on the analysis of clinical, laboratory and ultrasound data recorded in the HV‐FMVZ computerized system.

### 2.2 Inclusion Criteria

Dogs were included in the study if they presented with AKI or CKD in uraemic crisis, with no restrictions on age, weight or sex. The classification of dogs with CKD in uraemic crisis was based on the presence of uraemia with clinical signs such as hyporexia, anorexia, vomiting, weight loss and diarrhoea, as well as ultrasound changes indicating decreased renal corticomedullary differentiation, increased cortical echogenicity, decreased renal volume and irregular contour, or the presence of renal cysts (Bragato et al. 2017). Dogs were diagnosed with AKI if they presented with progressive azotaemia or uraemia, in the absence of a decrease in renal corticomedullary differentiation or renal cysts, with a regular renal contour and preserved or increased renal volume.

### 2.3 Exclusion Criteria

Based on clinical history and the results of complete blood count, serum biochemistry, venous blood gas and electrolyte analysis and abdominal ultrasonography performed prior to the haemodialysis sessions, dogs not in uraemic crisis, including those with hyperhydration or intoxication, were excluded. Dogs with prerenal or postrenal azotaemia were also excluded.

### 2.4 Collection of Blood Samples

Blood samples were obtained by jugular venipuncture five minutes before the start of the procedure and 5–30 min after the end of the dialysis session. Two millilitres of whole blood was used for serum biochemistry, 1 mL for haematology, and 1 mL for venous haemogasometry.

### 2.5 Haematology

Hematologic variables evaluated included erythrocyte count, haemoglobin, haematocrit, mean corpuscular volume (MCV), mean corpuscular haemoglobin concentration (MCHC), total plasma protein, total leukocyte count and differentiation and platelet count. Potassium EDTA tubes were used. One millilitre whole blood samples were analyzed within 30 min of collection (Advia 120 or 2120i, Siemens Medical Solutions Diagnostics).

Patients' activated clotting time (ACT) and anticoagulation data were also recorded before and every hour during the session until 30 min before the end of the procedure, as recommended by Cowgill (2011).

### 2.6 Serum Biochemistry

The results of the serum biochemistry evaluation included serum concentrations of urea, creatinine, total protein (PT), albumin, globulin and phosphorus. The 2 mL samples for serum biochemistry were collected in tubes with gel separators without anticoagulant, left to clot, centrifuged and the sera were collected and analyzed within 60 min of collection (Cobas Integra 400 Plus or Cobas 6000, Roche; at 37°C).

### 2.7 Venous Blood Gas and Electrolyte Analysis

Venous blood samples were used for blood gas analysis, hydrogen potential (pH), bicarbonate (HCO_3_), base excess (BE), anion gap, chloride, sodium, potassium, ionic calcium and lactate. Samples of 1 mL of venous blood were collected via syringes containing lithium heparin and analyzed within 10 min of collection (OmniC or Cobas b221, Roche).

### 2.8 Intermittent Haemodialysis

Regarding the platforms used, the IHD sessions at the dialysis centre between 2017 and 2024 were performed on the 4008S V10 platform (Fresenius Medical Care, Bad Homburg Höhe, Germany) coupled to a reverse osmosis water treatment unit (MCA.OR.PF.01, Palhoça, Santa Catarina, Brazil). Sessions were prescribed based on the calculation of URR as described by Cowgill (2011).

Over the years, capillary haemodialyzers with polysulphone membranes (Fresenius Medical Care, Bad Homburg Höhe, Germany) have been used according to body weight (<12 kg: 0.8 m^2^ dialyzers, 12–20 kg: 1.5 m^2^ dialyzers, over 30 kg: dialyzers larger than 2.0 m^2^ (Cowgill and Francey 2012). Sodium HCO_3_ buffer at 8.4% and an electrolyte solution with glucose made up the dialysis solution (Fresenius, Medical Care, Bad Homburg vor der Höhe, Germany). The final composition of the dialysis solution over the years included 145 mEq/L sodium, 3 mEq/L potassium, 35 mEq/L HCO_3_, 108.5 mEq/L chloride, 3.5 mEq/L calcium and 1 gL glucose diluted in ultrapure water. The urea removal rate (URR) was used to calculate the extracorporeal blood flow. The dialysate flow was maintained at 500 mL/min for all dogs (Bloom and Labato 2011).

The recommendations of Bloom and Labato (2011) were used to catheterize all patients. A double‐lumen catheter was placed in the right or left external jugular vein, with subsequent placement in the cranial vena cava or entry into the right atrium. Sedation was not used on any patient; only manual restraint was used.

Heparin sodium (Hemofol, Cristália Produtos Químicos Farmacêuticos Ltda, Itapira, São Paulo, Brazil) at an initial dose of 50 IU/kg and repeated every hour at the required dose was the drug of choice for anticoagulation in patients. If the activated clotting time (ACT) was between 1.6 and 2 times the normal value, or approximately 160–200 s, heparin administration was interrupted or the dose was reduced. Heparin was suspended 30 min before the end of the treatment (Bloom and Labato 2011).

### 2.9 Statistical Analysis

The assumption of normality was assessed visually and mathematically via the distribution of residuals and the Shapiro‒Wilk test, respectively. The means of the variables ACT and heparin dose over the days were compared via inference analysis with generalized linear mixed models (GLMMs) (Proc Glimmix). The covariance matrices were chosen via the Akaike's information criterion (AIC), consistent Akaike's information criterion (AICC) and Bayesian information criterion (BIC). The minute (M) was used as a fixed effect. The Pairwise Difference (PDIFF) command was used to compare the means adjusted with Tukey's post hoc test. The results are shown as the mean ± standard deviation (SD).

The variables that were normally distributed were analyzed via paired *t*‐tests or unpaired *t*‐tests, depending on the case. For variables that did not meet the assumption of a normal distribution, the tests of choice were the Wilcoxon or Mann–Whitney test, depending on the case. The results are shown as the mean ± standard deviation (SD) (parametric) or median (Q1‐Q3) (nonparametric). Statistical analysis was carried out via the SAS program (https://welcome.oda.sas.com/) and GraphPad Prism version 9.0.3.

Proportions were calculated with their respective 95% confidence intervals (95% CI) using the exact Clopper–Pearson method in R software (R Core Team, version 4.3.2)

## 3 Results

Fifty‐eight dogs and 149 sessions were included. The average body weight was 18.98 ± 10.09 kg, and the average age was 9.07 ± 4.21 years. A total of 53.45% of the dogs were male, and 46.55% were female. Mixed‐breed dogs were the most prevalent (51.72%; 30/58). Among purebred dogs, Labrador Retrievers were the most frequently represented (8.62%; 5/58), followed by Blue Heeler, Bull Terrier, Border Collie, Chow Chow, Dachshund, German Shepherd and Rottweiler (3.46% each). American Staffordshire Terrier, American Pit Bull Terrier, Beagle, Bulldog, Dogo Argentino, Great Dane, Belgian Shepherd, Shar Pei and Shih Tzu were each represented by one dog (1.72%).

Analysis of the blood count, serum biochemistry and blood gas data revealed a prevalence of anaemia, thrombocytopenia and hypoalbuminaemia in dogs in uraemic crisis at the time of admission for the first IHD session (Table 1). Among the anaemic dogs, 95.7% (95% CI: 85%–99%; 45/47) had normocytic normochromic anaemia, 2.1% (95% CI: 0.05%–11%; 1/47) had macrocytic and normochromic anaemia and another 2.1% (95% CI: 0.05%–11%; 1/47) had microcytic and normochromic anaemia.

**TABLE 1:** Frequency distribution (%) of erythrogram, thrombogram, albumin and serum pH abnormalities in dogs with uraemic crisis undergoing intermittent haemodialysis (IHD) at pre‐session time points, including all animals and those undergoing the first three sessions.

1st session (all dogs, *n* = 58)				1st to 3rd session (*n* = 22)					
Parameter	Interval	*n*	Anaemia	1st session		2nd session		3rd session	
				*n*	Anaemia	*n*	Anaemia	*n*	Anaemia
Haematocrit (%)	37–55^a^	11	81.03% (68%–90%)	4	81.8% (59%–94%)	1	95.4% (77%–99%)	0	100% (84%–100%)
	36–20	44		17		20		18	
	< 20	3		1		1		4	

1st session (all dogs, *n* = 48)				1st to 3rd session (*n* = 22)					
Parameter	Interval	*n*	Thrombocytopenia	1st session		2nd session		3rd session	
				*n*	Thrombocytopenia	*n*	Thrombocytopenia	*n*	Thrombocytopenia
Platelets (×10^4^/mL)	> 43	3	35.4% (22%–50%)	3		1		1	22.7% (7.8%–45%)
	16–43^a^	28		14	22.7% (7.8%–45%)	12	40.9% (20%–63%)	16	
	< 16	17		5		9		5	

1st session (all dogs, *n* = 48)				1st to 3rd session (*n* = 20)					
Parameter	Interval	*n*	Hypoalbuminaemia	1st session		2nd session		3rd session	
				*n*	Hypoalbuminaemia	*n*	Hypoalbuminaemia	*n*	Hypoalbuminaemia
Albumin (g/dL)	> 3.3	0	70.8% (55%–83%)	0		0		0	80% (56%–94%)
	2.6–3.3^a^	14		7	65% (40%–84%)	6	70% (45%–88%)	4	
	<2.6	34		13		14		16	

1st session (all dogs, *n* = 49)				1st to 3rd session (*n* = 22)					
Parameter	Interval	*n*	Acidaemia	1st session		2nd session		3rd session	
				*n*		*n*	Acidaemia	*n*	Acidaemia
pH	> 7.42	2	49% (33%–62%)	2		0		0	
	7.31–7.42^a^	23		12	36.4% (17%–59%)	16	27.3% (10%–50%)	20	9% (1.1%–29%)
	< 7.31	24		8		6		2	

^a^ Reference value Kaneko et al. (1997); Meyer and Harvey (2004); Weiss and Wardrop (2010).

When the underlying cause of uraemia (CKD or AKI) was analyzed, 84.6% (95% CI: 65%–95%; 22/26) of the dogs with AKI were anaemic at the time of admission to the IHD, with normocytic normochromic anaemia in 100% (95% CI: 84%–100%; 22/22) of the cases. Among patients with CKD, 78.1% (95% CI: 60%–90%; 25/32) had anaemia, with normocytic normochromic anaemia in 92% (95% CI: 73%–99%; 23/25) of cases.

No dogs received blood products during the haemodialysis sessions; however, 36.2% (95% CI: 23%–49%; 21/58) received blood products before or after at least one of the IHD sessions to which they were subjected. Among the dogs that received transfusions, 52.38% (95% CI: 29%–74%; 11/21) had CKD, whereas 47.61% (95% CI: 25%–70%; 10/21) had AKI.

Overall, 49% (95% CI: 3463%; 24/49) of the dogs had acidaemia, and 4% (95% CI: 0.04%–13%; 2/49) had alkalaemia. Among the patients with acidaemia, 91.7% (95% CI: 73%–98%; 22/24), corresponding to 44.89% (95% CI: 30%–59%; 22/49) of the 49 dogs with available pH values, had metabolic acidosis. One dog (1/24) had a mixed acid‒base disorder, and another (1/24) had respiratory acidosis. The alkalosis was exclusively respiratory in 100% (2/2) of the patients with alkalaemia. Among the patients with metabolic acidosis, 72.7% (95% CI: 49%–89%; 16/22) had a concomitant reduction in HCO_3_ and PCO_2_, whereas 27.3% (95% CI: 10%–50%; 6/22) only had a reduction in HCO_3_. Among those with a pH within the reference range, 43.5% (95% CI: 23%–65%; 10/23) showed a decrease in HCO_3_ and PCO_2_. The prevalence of metabolic acidosis on admission to the IHD was 40.6% (95% CI: 24%–59%; 13/32) in patients with CKD and 34.6% (95% CI: 17%–55%; 9/26) in those with AKI. Among patients with metabolic acidosis and AKI, 88.9% (95% CI: 51%–99%; 8/9) had a concomitant reduction in HCO_3_ and PCO_2_, whereas this configuration was observed in 53.8% (95% CI: 25%–80%; 7/13) of patients with CKD.

In addition, there were significant reductions in the concentrations of RBC, haemoglobin, MCHC, total protein, platelets, eosinophils, creatinine, urea, phosphorus, PT, albumin, globulin, haematocrit in venous blood gas analysis, BE and potassium after the IHD sessions. On the other hand, significant increases in the concentrations of MCV, RDW, pH, HCO_3_, the anion gap, chloride, sodium and ionized calcium were detected (Tables 2, 3, 4).

**TABLE 2:** Mean (standard deviation = SD) or median (Q1–Q3) haematological parameters before (pre) and after (post) dialysis in dogs with uraemic crisis undergoing intermittent haemodialysis (IHD) Kaneko et al. (1997); Meyer and Harvey (2004); Weiss and Wardrop (2010).

		Moments			
Variable	Reference value	Pre	Post	*n*	*p*
**Mean (±SD)**					
Red blood cells (×10^6^/mL)	5.5–8.5	3.9(±1.17)^a^	3.33(±0.96)^b^	135	< 0.0001
Haemoglobin (g/dL)	12–18	9.15(±2.61)^a^	7.85(±2.13)^b^	142	< 0.0001
Haematocrit (%)	37–55	26.8(±7.27)^a^	23.38(±6.29)^b^	144	< 0.0001
MCV (fL)	60–77	68.63(±5.34)^b^	70.19(±5.26)^a^	135	< 0.0001
Total protein (mg/dL)	6–8	7.32(±0.94)^a^	6.29(±0.79)^b^	142	< 0.0001
**Median (Q1–Q3)**					
MCHC (%)	31–36	33.80(32.7–35.2)^a^	33.40(32.6–34.48)^b^	136	0.001
RDW (%)	12–15	13.75(12.83–14.7)^b^	13.90 (13.13–14.80)^a^	104	0.004
Platelets (/mm^3^)	160,000–430,000	226,000 (145,588–279,000)^a^	129,000 (88,000–203,500)^b^	110	< 0.0001
Leukocytes (/mm^3^)	6000–17,000	11,750(8750–18,125)^a^	12,350(8975–17,325)^a^	142	0.47
Lymphocytes (/mm^3^)	1000–4800	600(300–1100)^a^	600(400–1000)^a^	141	0.7
Eosinophils (/mm^3^)	100–1250	100(0–350)^a^	100(0–300)^b^	141	0.009
Segmented neutrophils (/mm^3^)	3000–11,500	10,400(6800–15,750)^a^	10,900(7650–15,300)^a^	141	0.23
Monocytes (/mm^3^)	150–1350	600(300–1100)^a^	500(300–1000)^a^	141	0.39

Abbreviations: MCHC, mean corpuscular haemoglobin concentration; MCV, mean corpuscular volume.

^a, b^Different letters on the same line indicate a significant difference (*p* < 0.05) between the values before (pre) and after (post) the intermittent haemodialysis (IHD) session.

**TABLE 3:** Mean (standard deviation = SD) or median (Q1–Q3) serum biochemistry parameters before (pre) and after (post) dialysis in dogs with uraemic crisis undergoing intermittent haemodialysis (IHD) Kaneko et al. (1997); Meyer and Harvey (2004); Weiss and Wardrop (2010).

		Moment			
Variable	Reference value	Pre	Post	*n*	*p*
**Mean (±SD)**					
Urea (mg/dL)	21.4–59.92	360.4(±157.5)^a^	166.4(±77.64)^b^	146	< 0.0001
Creatinine (mg/dL)	0.5–1.5	9.693(±4.95)^a^	4.94(±3.02)^b^	144	< 0.0001
Phosphorus (mg/dL)	2.6–6.2	13.6(±4.87)^a^	6.163(±2.51)^b^	123	< 0.0001
Total calcium (mg/dL)	9–11.3	8.12(±2.54)^a^	7.74(±1.16)^a^	34	0.27
**Median (Q1–Q3)**					
PT (g/dL)	5.4–7.1	5.6(5.02–6.3)^a^	5.1(4.52–5.77)^b^	80	< 0.0001
Albumin (g/dL)	2.6–3.3	2.3(2–2.6)^a^	2.2(1.9–2.4)^b^	109	< 0.0001
Globulin (g/dL)	2.7–4.4	3.25(2.72–3.8)^a^	2.95(2.4–3.47)^b^	76	< 0.0001

^a, b^Different letters on the same line indicate a significant difference (*p* < 0.05) between the values before (pre) and after (post) the intermittent haemodialysis (IHD) session.

**TABLE 4:** Mean (standard deviation = SD) or median (Q1–Q3) venous blood gas analysis parameters before (pre) and after (post) dialysis in dogs with uraemic crisis undergoing intermittent haemodialysis (IHD) Vanova‐Uhrikova et al. (2017); Kaneko et al. (1997); Meyer and Harvey (2004); Weiss and Wardrop (2010).

		Moment			
Variable	Reference value	Pre	Post	*n*	*p*
**Mean (±SD)**					
HCO_3_ (mg/dL)	18–24	16.09(±3.54)^b^	21.27(±3.25)^a^	116	< 0.0001
Chloride (mg/dL)	105–115	107.4(±5.91)^b^	110.69(±3.59)^a^	114	< 0.0001
Potassium (mEq/L)	4.37–5.65	4.42(±0.91)^a^	3.894(±0.48)^b^	115	< 0.0001
**Median (Q1–Q3)**					
pH	7.31–7.42	7.33(7.29–7.37)^a^	7.41(7.37–7.44)^b^	115	< 0.0001
Haematocrit (%)	37–45	28(23–33.5)^a^	23(18–28)^b^	109	< 0.0001
BE	−4.5–0.3	9(5.7–11.25)^a^	2.5(1.25–4.3)^b^	113	< 0.0001
Anion	13–18	17(21.3–29.70)^b^	24.70 (14.7–20.6)^a^	117	< 0.0001
Sodium (mEq/L)	141.1–157	149(146–151)^b^	150(147–152)^a^	113	0.043
Ionic calcium (mEq/L)	1.16–1.4	0.99(0.77–1.21)^b^	1.15(0.95–1.25)^a^	109	< 0.0001
Lactate (mmol/L)	0.5–2.5	1(0.65–1.4)^a^	1(0.6–1.3)^a^	93	0.37

^a, b^Different letters on the same line indicate a significant difference (*p* < 0.05) between the values before (pre) and after (post) the intermittent haemodialysis (IHD) session.

**FIGURE 1:**
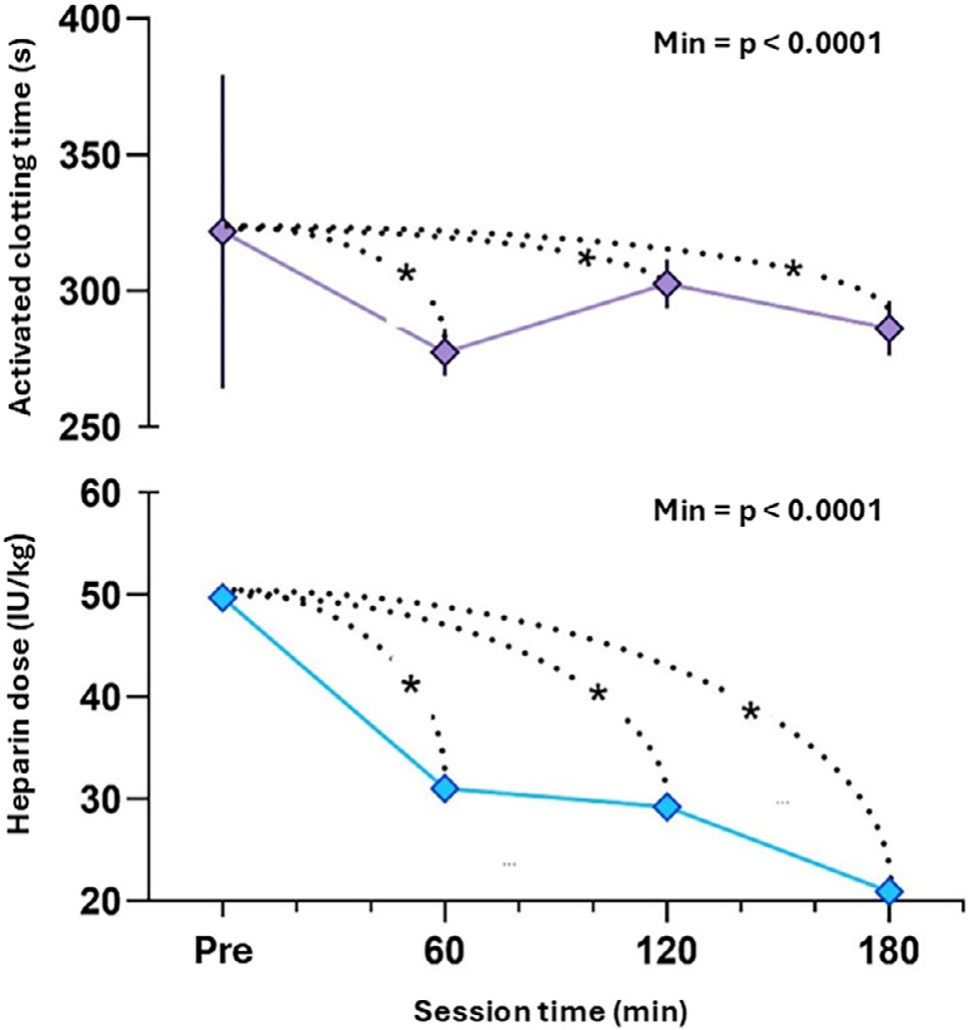
Temporal variation in activated clotting time (s) and sodium heparin dose (IU/kg) in uraemic dogs undergoing intermittent haemodialysis (IHD). Significant difference (*p* < 0.05).

The assessment of ACT and heparin dose, which are dependent variables, revealed significant variation over time in the IHD (Figure 1 and Table 5). The number of patients with ACT and heparinized differs due to the absence of data in the medical records on the dose administered. However, all patients received heparin. Clot formation was observed in 2 of 149 sessions (1.34%; 95% CI: 0.16%–4.7%).

**TABLE 5:** Mean (standard deviation = SD) of activated clotting time (s) and dose of heparin sodium (IU/kg) in dogs with uraemic crisis undergoing intermittent haemodialysis (IHD).

Activated clotting time (ACT) (s) and heparin dose (IU/kg)									
Time (min)	Pre		60		120		180		
Variable	TCA	Heparin	TCA	Heparin	TCA	Heparin	TCA	Heparin	Target value^c^
Average	321.7^a^	49.7^a^	277.4b	31^b^	302.5^b^	29.2^b^	286.2^b^	20.9^b^	1.6–2 (baseline value)
Standard deviation	61	6	98.59	12.23	150.29	11.51	80.66	12.26	
*n*	138	122	134	109	124	92	99	75	

Abbreviation: *n*, sample size.

^a, b^Different superscript letters on the same line indicate a significant difference (*p* < 0.0001) between the moments.

^c^Bloom and Labato (2011).

## 4 Discussion

This study revealed that, upon admission to the IHD, dogs in uraemic crisis typically exhibit a laboratory profile characterized by a high prevalence of normocytic normochromic anaemia, thrombocytopenia and hypoalbuminaemia. A progressive decrease in serum albumin concentration and the possibility of anaemia developing in up to 100% of patients have been demonstrated with an increasing number of dialysis treatments. The average decrease in haematocrit per session was also estimated, aiding in the prediction of blood losses associated with IHD, supporting both the selection of priming fluids and the evaluation of the need for blood product replacement. A high prevalence of acidaemia and metabolic acidosis with compensatory respiratory alkalosis, and a high prevalence of physiological pH with decreased HCO_3_ levels accompanied by respiratory alkalosis, were observed on admission to the IHD. This suggests that compensatory respiratory alkalosis may modulate pH to remain within the reference range in a proportion of uraemic crisis dogs until correction through dialysis is implemented.

Normocytosis and normochromia in renal patients admitted to the IHD reflect the high prevalence of normocytic normochromic anaemia in the population of dogs with CKD and AKI (King et al. 1992; Lippi et al. 2021). This classification is relevant for guiding strategies aimed at mitigating non‐regenerative anaemia in this population, given the high prevalence found. Causes include chronic blood loss, particularly associated with gastrointestinal bleeding (overt or occult); erythropoietic hypoproliferation; and the action of inflammatory cytokines, aggravated by the accumulation of uraemic toxins (Harris and Krawiec 1990; Crivellenti et al. 2017; Borin‐Crivellenti et al. 2023).

On the other hand, non‐chronic anaemia factors, such as the onset of infectious and inflammatory diseases, which often cause an abrupt loss of renal function (Rimer et al. 2022), may not allow enough time for the medullary response to develop properly in the face of progressive azotaemia or uraemia. This may explain the normocytic normochromic profile observed in 100% of the AKI patients in this study. In addition, AKI can drastically reduce erythropoietin production, and the critical conditions associated with this acute condition often result in resistance to the action of endogenous erythropoietin, compromising the replacement of lost or damaged RBC (Liangos et al. 2003; Prakash 2012).

When considering those animals that received at least three IHD sessions, the prevalence of anaemia increased with the number of treatments, possibly due to the use of anticoagulants and acute blood loss in the extracorporeal circuit, which exacerbates the aforementioned causes of RBC destruction or loss (Langston et al. 1997; Langston et al. 2017). King et al. (1992) found a direct correlation between the severity of anaemia and the progression of renal disease, as evidenced by an increase in creatinine levels, a relationship also confirmed by Borin‐Crivellenti et al. (2023). Thus, the decrease in haematocrit associated with the loss of renal function may explain the higher prevalence of anaemia (92%) observed in patients undergoing IHD in this study, compared to the 76.47% reported by King et al. (1992) in their clinical trial, which included patients not treated with dialysis, suggesting less severe clinical conditions. This increase in prevalence is particularly relevant, as anaemia significantly affects the quality of life of animals with CKD (Polzin 2011), and, consequently, those undergoing haemodialysis.

In this context, blood transfusions may be necessary. Langston et al. (2017) reported a need for transfusion in at least 32% of dogs treated with IHD. The average reduction of 3.42 percentage points in haematocrit (*p* < 0.0001; *n* = 144) measured by complete blood count and 5 percentage points in haematocrit obtained by venous blood gas analysis (*p* < 0.0001; *n* = 109) after IHD sessions in this study allows for early adjustments in prescriptions, especially for dogs with haematocrit close to 20%, which is considered a critical point in the management of anaemic patients during haemodialysis (Segev et al. 2024). The average reduction in haematocrit observed helps in the choice of priming solutions with plasma volume‐expanding properties, which are essential for maintaining vascular homeostasis (Segev et al. 2024). An average 5% reduction in the haematocrit was also reported by Meneses et al. (2010) in their clinical trial.

Furthermore, the high prevalence of hypoproteinaemia in the dogs before the first IHD session and its intensification over the course of the treatments observed in this retrospective study may be explained by protein losses common in patients with kidney damage, such as proteinuria and cachexia (Pérez‐Sánchez et al. 2023). Additional protein losses can occur in the dialyzer, depending on its flow characteristics.

Metabolic acidosis was a common condition in this study. Renal dysfunction compromises the excretion of hydrogen ions and the reabsorption of HCO_3_, creating tubular conditions conducive to the development of acidaemia (Palmer et al. 2021). In patients undergoing IHD due to uraemic crisis, the inclusion criterion for this study, these alterations become even more likely, given the severity of the deregulation of renal physiology, a reflection of advanced‐stage CKD or severe uraemia resulting from the abrupt interruption of renal function. Moranne et al. (2009) demonstrated an increase in the prevalence of metabolic acidosis in humans as the glomerular filtration rate decreases, corroborating the above findings.

Although uraemia contributes to systemic dysfunctions, the high prevalence of metabolic acidosis at admission on IHD, associated with a decrease in serum HCO_3_ and PCO_2_ concentrations in the dogs in this study, suggests the activation of compensatory mechanisms typical of acid‐base homeostasis, as described by Hamm et al. (2015). A slight decrease in plasma and cerebral interstitial pH stimulates an increase in alveolar ventilation, resulting in a decrease in PCO_2_ (Hamm et al. 2015). However, this respiratory alkalosis secondary to metabolic acidosis was not sufficient to normalize the pH in these dogs, which remained outside the reference range despite compensation. The authors believe that this regulation was limited both by the insufficient time for the compensatory response and by the inefficiency of these mechanisms in attenuating pH changes in the context of the severity of kidney disease. This aspect is relevant, as metabolic acidosis contributes to additional kidney dysfunction, as well as being, associated with bone deterioration, muscle loss and progression of kidney disease (Chen and Abramowitz 2014; Raphael 2018), making IHD beneficial for correcting this disorder.

On the other hand, compensation for metabolic acidosis through the development of respiratory alkalosis has proven to be efficient in at least 43.5% of dogs, among those with normal pH, due to the concomitant reduction in serum HCO_3_ and PCO_2_ concentrations. A normal pH, associated with a reduction in HCO_3_ and PCO_2_, suggests an effective self‐regulatory mechanism for mitigating acidaemia (Hamm et al. 2015) until therapeutic measures, such as HCO_3_ supplementation or dialysis, can be implemented.

Finally, the significant reductions in the concentrations of RBC, haemoglobin, haematocrit, MCHC, platelets, creatinine, urea, phosphorus, PT, albumin, globulin, BE, anion gap and potassium observed in this study have been previously reported by other authors (Meneses et al. 2010; Geraldes et al. 2019). The reduction in RBC and platelet levels may be associated with RBC and platelet destruction during blood circulation through the extracorporeal circuit, the use of heparin, blood loss and uraemic and haemodialysis‐induced inflammation. (Langston et al. 2017; Picelli et al. 2022; Borin‐Crivellenti et al. 2023).

The significant reduction in mean ACT values during haemodialysis reinforces the importance of serial ACT monitoring, as recommended by Bloom and Labato (2011) and Segev et al. (2024). The observed reduction can be attributed to activation of the coagulation cascade resulting from blood contact with the extracorporeal circuit, a condition that requires effective anticoagulation to prevent clot formation (Bloom and Labato 2011). The low number of intradialytic coagulation‐related events suggests that the heparin doses administered and the ACT levels achieved were adequate for the hemostatic management of the animals throughout the evaluated period. These findings reinforce that anticoagulation protocols should be evaluated individually, considering the haemostatic particularities of each patient and their respective heparin dose requirements.

The main limitations of this study include the lack of data due to incomplete medical records, which resulted in discrepancies in the number of animals or sessions analyzed for each variable.

In conclusion, the laboratory alterations observed allow early therapeutic planning for patients undergoing IHD. The high prevalence of anaemia and hypoalbuminaemia upon admission to IHD, with a progressive increase in these conditions as the number of sessions increases, emphasizes the need for rigorous laboratory monitoring. The high prevalence of acidaemia, metabolic acidosis, and secondary respiratory alkalosis, along with the reduction in their prevalence after dialysis treatment, contributes to the characterization of the acid‐base profile of dogs in uraemic crisis admitted to dialysis centres, reaffirming the efficacy of IHD as a therapeutic method for correcting these disorders. In addition, the estimated mean reductions of 3.42‐5% in haematocrit% per session allow for more accurate therapeutic and operational planning, supporting the selection of priming fluid and the assessment of the need for blood product replacement.

## Author Contributions

**Diego Ribeiro**: conceptualization, methodology, data curation, data collection, formal analysis, investigation, resources, writing – original draft, writing – review and editing. **Reiner S. de Moraes**: data collection, investigation, resources. **Silvano S. Geraldes**: data collection, investigation, resources. **Henry D. Mogollon‐García**: data curation, formal analysis, methodology, software. **Paulo F. Marcusso**: conceptualization, methodology, data curation, formal analysis, investigation, supervision, writing – review and editing. **Alessandra Melchert**: conceptualization, methodology, data curation, formal analysis, investigation, supervision, writing – review and editing. **Adriano S. Okamoto**: conceptualization, methodology, data curation, formal analysis, investigation, supervision, writing – review and editing. **Priscylla T. C. Guimarães‐Okamoto**: conceptualization, methodology, data curation, formal analysis, investigation, supervision, writing – review and editing.

## Funding

The authors have nothing to report.

## Ethics Statement

This research was conducted in accordance with the recommendations of the National Institutes of Health's Guide for the Care and Use of Laboratory Animals. The protocol was approved by the Animal Experiments Ethics Committee of the São Paulo State University (UNESP) (Protocol Number: 000.116/2024).

## Conflicts of Interest

The authors declare no conflicts of interest.

## Data Availability

All data supporting the conclusions of this study are included in the manuscript.
